# Supplementary material for: Immunolocalization of the AT-1R Ang II Receptor in Human Kidney Cancer
Source: Biomolecules. 2023 Jul 28;13(8):1181. doi: 10.3390/biom13081181 (PMC10452411; doi:10.3390/biom13081181)
Supplement: Supplementary file 1 [file biomolecules-13-01181-s001.zip › biomolecules-2311320- Figure S1.pdf]

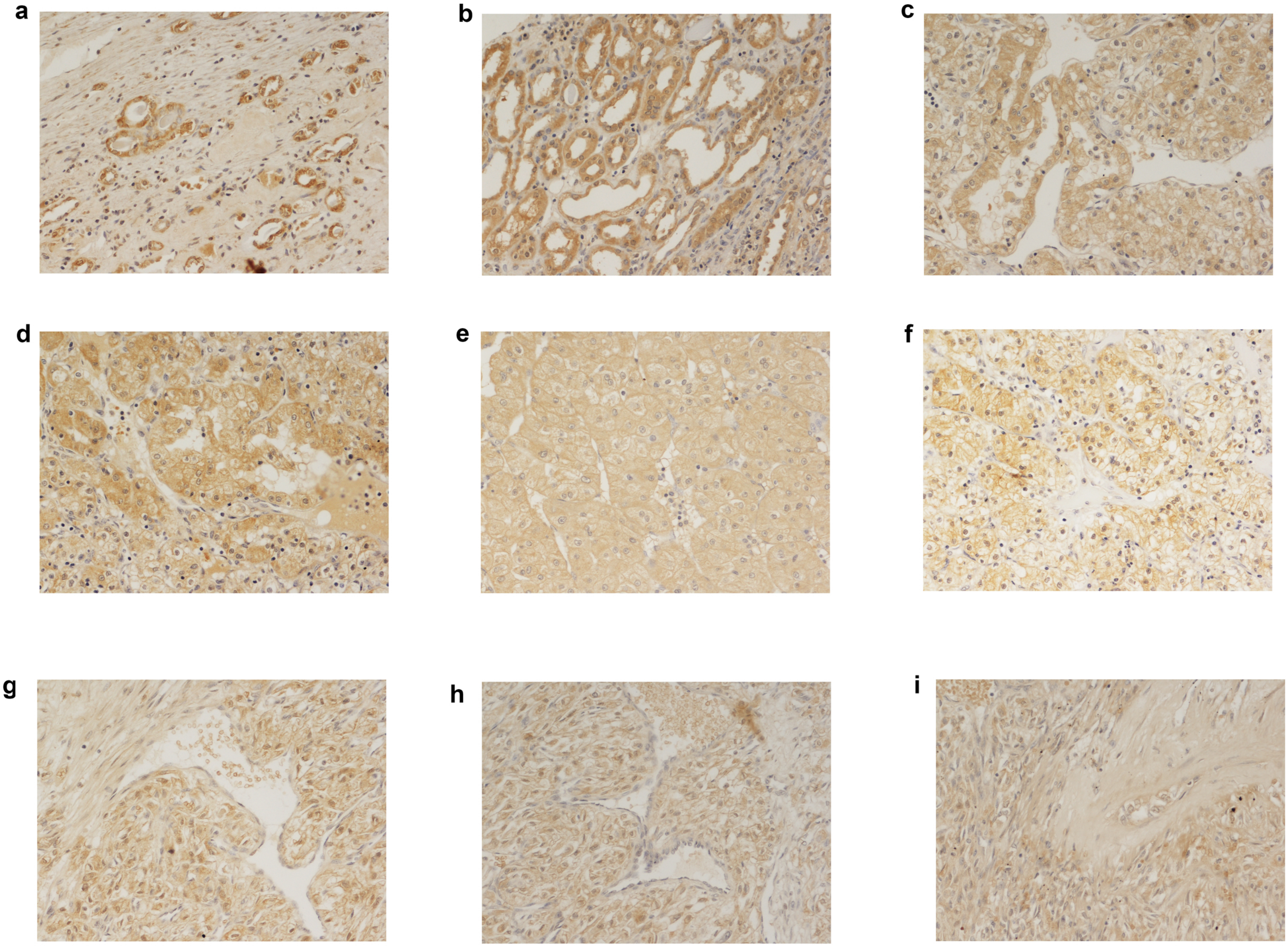

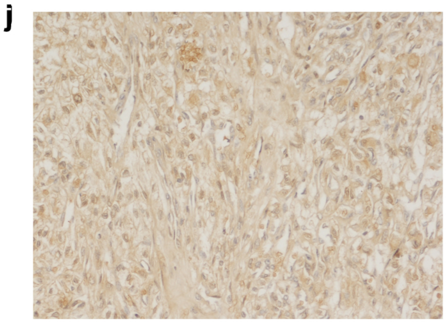

h

| | G1 | G2 | G3 | G4 |
|---|---|---|---|---|
| **Sum of the grey level values Mean ± S.D. (x10$^6$)** | 6.6 ± 0.7 | 4.2 ± 0.7 | 2.1 ± 0.6 | 1.4 ± 0.7 |

**Figure S1. The expression of AT-1R in normal kidney and cancer tissues immunostaining of paraffin-embedded human kidney**

**a, b,** AT-1R is expressed in normal proximal tubule cells.

**c, d, e,** well-differentiated carcinomas (G1); f, moderately differentiated carcinomas (G2)

**g - j,** poorly differentiated carcinomas (g, h,G3; i,j G4).

**h,** The sum of grey levels obtained by image analysis performed on tumoral kidney tissues classified in G1, G2, G3, and G4. Values refer to the mean data ± S.D. from 10 analyzed randomly chosen microscopic fields, of each section.
