# Supplementary material for: Immunolocalization of the AT-1R Ang II Receptor in Human Kidney Cancer
Source: Biomolecules. 2023 Jul 28;13(8):1181. doi: 10.3390/biom13081181 (PMC10452411; doi:10.3390/biom13081181)

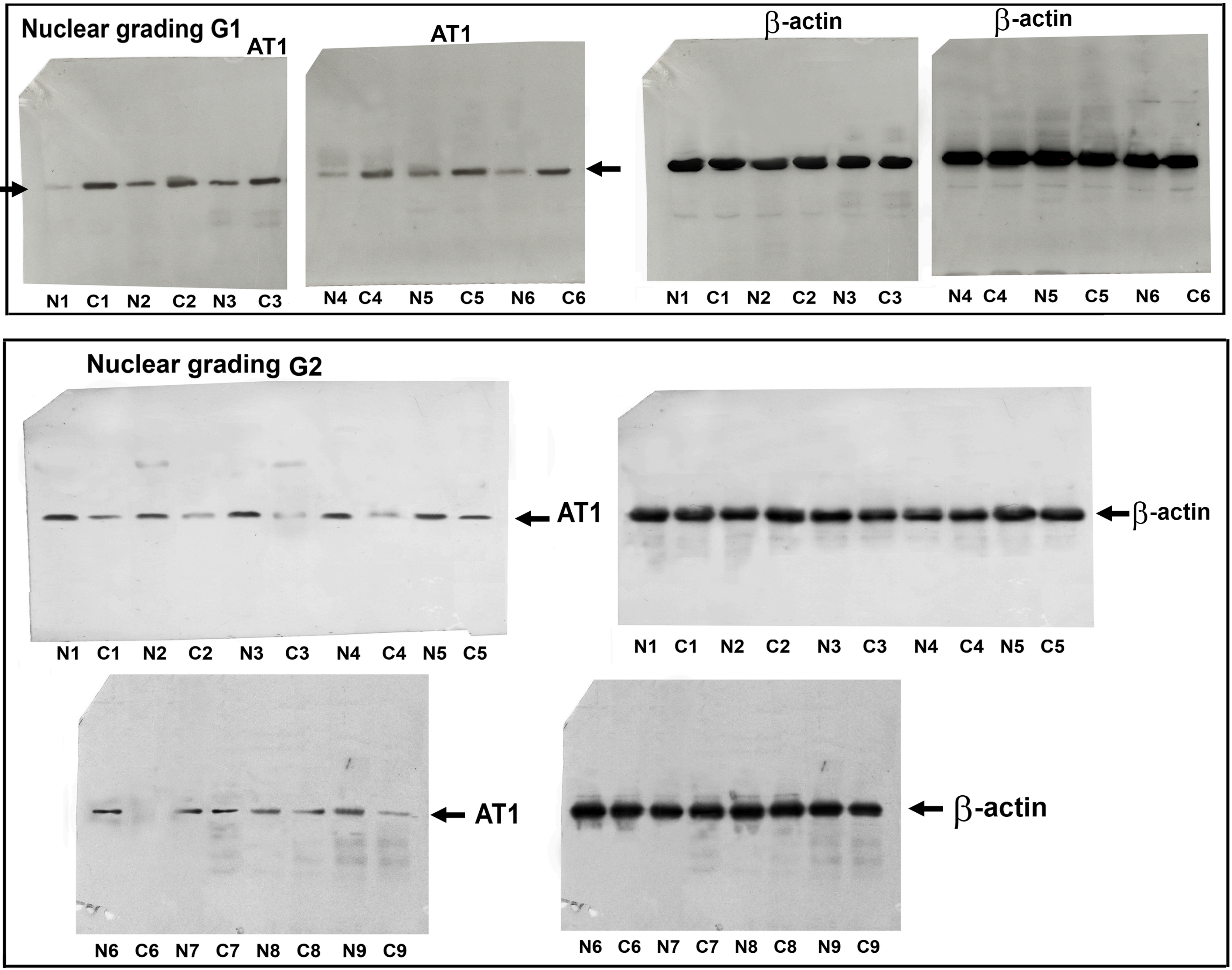
Nuclear grading G1
AT1
AT1
β-actin
β-actin
N1 C1 N2 C2 N3 C3
N4 C4 N5 C5 N6 C6
N1 C1 N2 C2 N3 C3
N4 C4 N5 C5 N6 C6
Nuclear grading G2
AT1
β-actin
N1 C1 N2 C2 N3 C3 N4 C4 N5 C5
N1 C1 N2 C2 N3 C3 N4 C4 N5 C5
AT1
β-actin
N6 C6 N7 C7 N8 C8 N9 C9
N6 C6 N7 C7 N8 C8 N9 C9

Nuclear grading G3

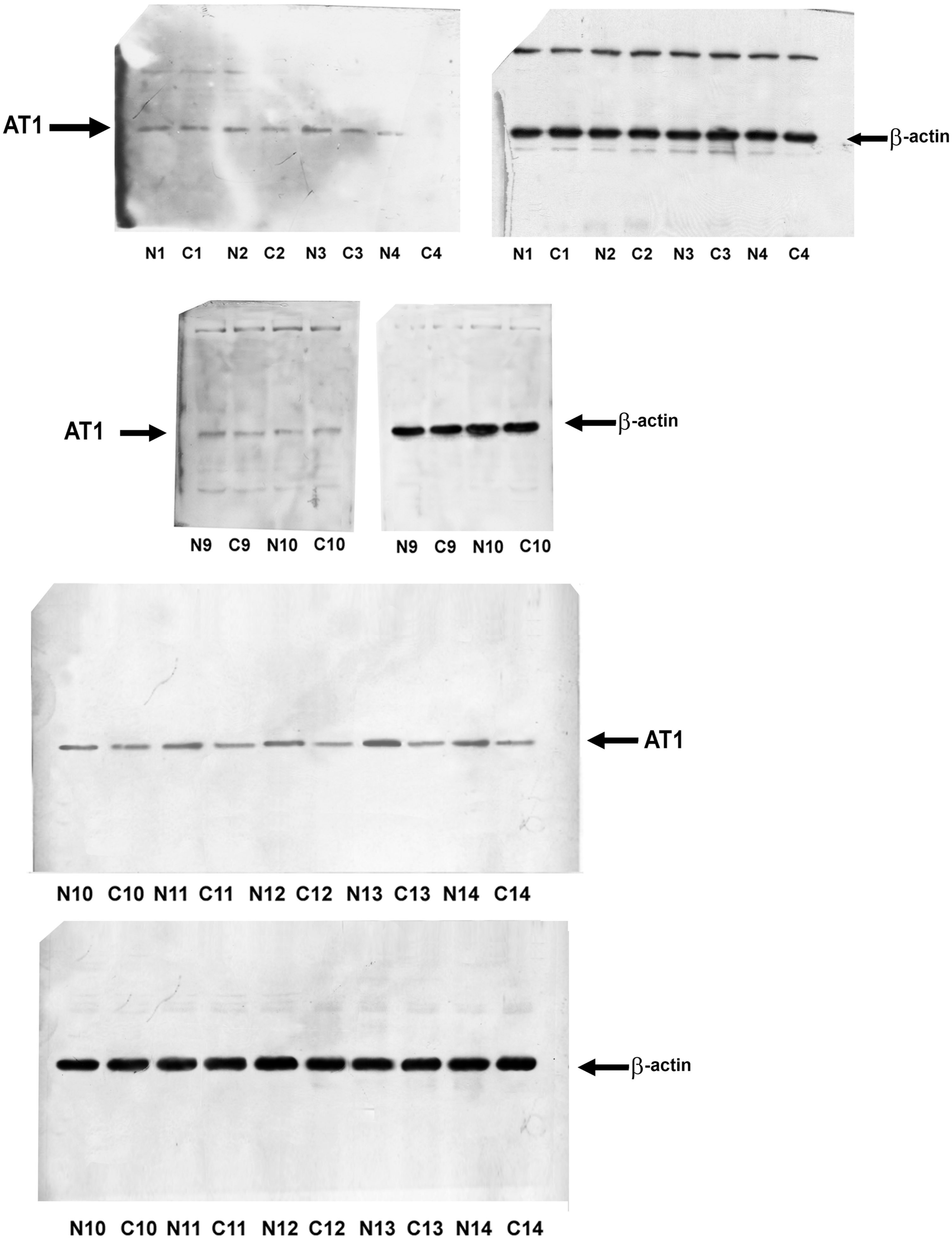

Nuclear grading G4

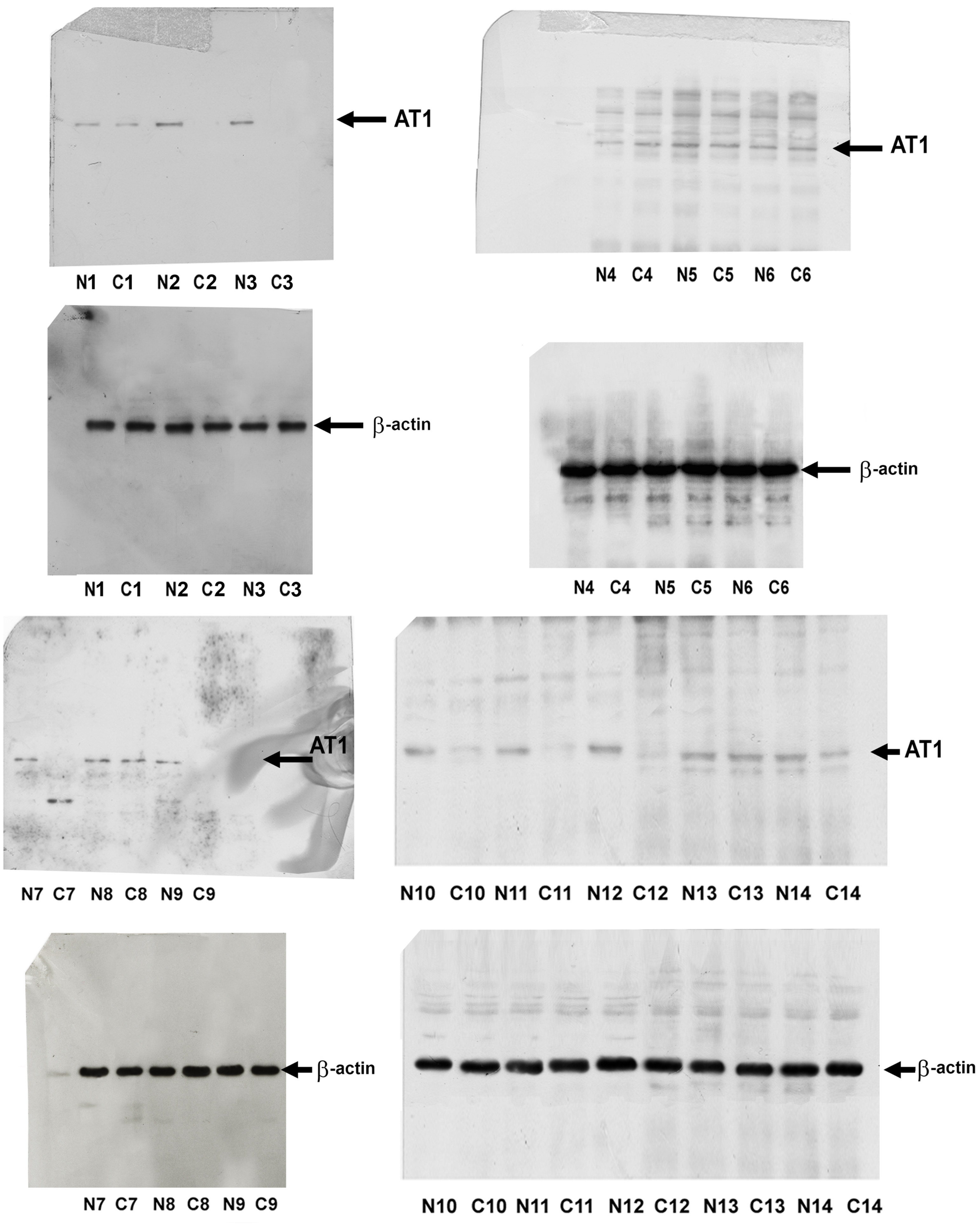

Supplement: Supplementary file 1 [file biomolecules-13-01181-s001.zip › biomolecules-2311320- Supplementary S1.pdf]
