# Supplementary material for: Immunolocalization of the AT-1R Ang II Receptor in Human Kidney Cancer
Source: Biomolecules. 2023 Jul 28;13(8):1181. doi: 10.3390/biom13081181 (PMC10452411; doi:10.3390/biom13081181)
Supplement: Supplementary file 1 [file biomolecules-13-01181-s001.zip › biomolecules-2311320- Table S1.pdf]

Table S1. Densiometric quantification of the AT-1R and β-actin bands obtained by immunoblotting analysis performed on 40 normal (N) and 42 tumoral kidney tissues classified in G1, G2, G3 and G4. For each sample three values are reported which correspond to the densitometries obtained on the same samples loaded in triplicate onto different gels.

Specimen	G1						G2						G3						G4					
	N			T			N			T			N			T			N			T		
	AT1	actin	Ratio	AT1	actin	Ratio	AT1	actin	Ratio	AT1	actin	Ratio	AT1	actin	Ratio	AT1	actin	Ratio	AT1	actin	Ratio	AT1	actin	Ratio
1	34,661	80,234	0,432	61,772	82,253	0,751	41,575	82,655	0,503	21,268	84,398	0,252	38,562	75,463	0,511	4,344	85,012	0,051	31,456	82,345	0,382	1,702	75,398	0,023
	35,222	81,344	0,433	62,859	81,319	0,773	40,658	81,643	0,498	22,084	83,970	0,263	37,522	74,745	0,502	4,299	84,630	0,051	34,534	84,023	0,411	1,584	75,455	0,021
	34,753	79,345	0,438	54,088	82,830	0,653	40,190	78,649	0,511	21,556	83,876	0,257	37,281	75,467	0,494	4,394	87,347	0,050	32,619	81,345	0,401	1,190	76,044	0,016
2	27,006	84,132	0,321	53,337	69,997	0,762	35,194	83,399	0,422	34,105	79,872	0,427	45,616	73,456	0,621	3,024	76,569	0,039	39,484	83,124	0,475	0,000	71,877	0,000
	28,500	83,333	0,342	61,181	75,254	0,813	35,963	82,675	0,435	34,256	79,113	0,433	45,015	75,275	0,598	3,004	77,213	0,039	46,526	84,439	0,551	0,000	72,984	0,000
	26,685	84,180	0,317	55,483	74,775	0,742	36,775	85,127	0,432	32,780	77,863	0,421	46,040	72,964	0,631	2,947	75,963	0,039	42,516	84,024	0,506	0,000	72,126	0,000
3	37,774	75,398	0,501	74,581	83,238	0,896	17,020	75,645	0,225	8,550	82,988	0,103	39,009	83,353	0,468	3,019	73,456	0,041	36,152	70,887	0,510	0,000	82,593	0,000
	38,169	74,548	0,512	79,618	84,251	0,945	18,507	73,735	0,251	10,011	81,813	0,122	39,630	84,861	0,467	3,224	74,638	0,043	36,966	72,769	0,508	0,000	84,132	0,000
	38,706	76,044	0,509	74,174	81,153	0,914	17,558	76,675	0,229	9,534	80,798	0,118	40,050	84,673	0,473	3,084	73,776	0,042	36,608	72,780	0,503	0,001	83,869	0,000
4	24,798	71,877	0,345	46,518	84,272	0,552	43,713	72,612	0,602	23,804	73,696	0,323	24,233	70,651	0,343	0,003	83,399	0,000	22,800	82,908	0,275	2,923	75,445	0,039
	25,909	72,984	0,355	45,358	81,873	0,554	44,403	74,377	0,597	24,815	74,744	0,332	24,625	72,854	0,338	0,003	82,675	0,000	22,136	84,813	0,261	2,919	73,667	0,040
	24,650	70,630	0,349	45,856	83,223	0,551	45,143	73,643	0,613	26,961	73,866	0,365	24,877	72,954	0,341	0,001	85,127	0,000	20,165	80,983	0,249	3,142	76,567	0,041
5	40,481	83,295	0,486	60,395	75,969	0,795	39,448	82,527	0,478	20,998	82,345	0,255	23,067	82,977	0,278	4,939	80,435	0,061	36,725	85,012	0,432	2,568	72,546	0,035
	41,414	86,639	0,478	57,201	78,143	0,732	39,315	84,186	0,467	21,342	84,023	0,254	22,730	84,813	0,268	4,858	81,234	0,060	36,814	84,630	0,435	2,498	74,574	0,033
	38,545	82,537	0,467	59,001	76,426	0,772	38,371	81,295	0,472	24,587	83,345	0,295	22,432	80,983	0,277	4,883	79,789	0,061	38,171	87,347	0,437	2,508	73,126	0,034
6	32,377	82,593	0,392	71,796	83,873	0,856	23,194	69,651	0,333	0,004	83,124	0,000	40,891	85,012	0,481	4,282	82,670	0,052	37,978	76,569	0,496	1,768	82,215	0,022
	40,468	84,132	0,481	66,529	84,320	0,789	24,776	75,537	0,328	0,002	84,439	0,000	40,199	84,630	0,475	4,431	84,568	0,052	36,889	77,012	0,479	1,866	84,432	0,022
	32,027	79,869	0,401	70,153	82,147	0,854	25,419	74,542	0,341	0,003	85,024	0,000	50,486	87,347	0,578	4,280	83,760	0,051	39,490	75,796	0,521	1,674	81,673	0,021
7							23,119	83,765	0,276	8,861	70,887	0,125	25,497	76,569	0,333	3,857	74,890	0,051	19,078	79,946	0,239	0,000	69,777	0,000
							22,379	84,133	0,266	11,842	71,769	0,165	26,484	77,213	0,343	3,845	75,238	0,051	20,516	83,399	0,246	0,000	75,455	0,000
							22,859	81,348	0,281	13,246	72,780	0,182	24,840	75,963	0,327	3,905	74,946	0,052	20,834	82,675	0,252	0,000	74,542	0,000
8							41,335	84,185	0,491	9,281	82,135	0,113	29,823	73,456	0,406	3,617	83,733	0,043	43,670	85,127	0,513	3,713	83,124	0,045
							38,615	81,295	0,475	10,373	84,330	0,123	30,975	74,638	0,415	3,778	84,719	0,045	38,311	75,565	0,507	3,827	82,227	0,047
							45,924	83,347	0,551	10,209	81,673	0,125	30,470	73,776	0,413	3,391	82,295	0,041	37,796	73,677	0,513	3,619	84,127	0,043
9							19,347	75,872	0,255	17,804	79,128	0,225	19,003	83,346	0,228	3,951	82,135	0,048	25,520	76,867	0,332	0,001	75,565	0,000
							19,997	78,113	0,256	19,539	77,846	0,251	18,993	84,041	0,226	3,804	84,202	0,045	24,515	72,961	0,336	0,000	74,868	0,000
							18,007	76,626	0,235	17,769	79,325	0,224	18,597	82,653	0,225	3,798	81,335	0,047	24,418	74,444	0,328	0,003	76,867	0,000
10													31,209	80,435	0,388	3,479	83,424	0,042	16,574	73,664	0,225	4,001	79,801	0,050
													32,575	81,234	0,401	3,602	84,544	0,043	16,529	82,235	0,201	4,128	89,987	0,046
													32,235	79,789	0,404	3,689	84,230	0,044	17,163	84,132	0,204	4,202	80,964	0,052
11													43,227	82,653	0,523	3,183	70,889	0,045	35,384	81,343	0,435	5,016	82,235	0,061
													48,180	80,435	0,599	3,307	72,678	0,045	30,195	69,897	0,432	5,149	84,132	0,061
													45,328	81,234	0,558	3,148	72,878	0,043	32,042	75,215	0,426	4,921	81,343	0,061
12													33,496	73,456	0,456	2,687	82,932	0,032	35,732	74,910	0,477	3,970	69,897	0,057
													35.831	75,275	0,476	2,927	84,343	0,035	40,667	83,677	0,486	4,347	75,215	0,058
													34,658	72,964	0,475	3,083	81,983	0,038	41,425	84,541	0,490	4,262	74,910	0,057
13													42,161	82,345	0,512	3,601	83,346	0,043				3,146	83,677	0,038
													40,509	81,345	0,498	3,622	84,041	0,043				3,149	84,541	0,037
													40,564	83,124	0,488	3,695	82,653	0,045				3,046	81,653	0,037
14																						1,652	84,432	0,020
																						1,558	81,763	0,019
																						1,538	83,543	0,018
**Means**	33,442	79,617	**0,420**	61,105	80,295	**0,761**	31,808	79,158	**0,401**	20,111	79,700	**0,251**	30,024	78,933	**0,432**	3,693	80,603	**0,042**	31,899	79,585	**0,402**	3,053	78,651	**0,027**
